# ^64^Cu-DOTATATE Positron Emission Tomography (PET) of *Borrelia Burgdorferi* Infection: In Vivo Imaging of Macrophages in Experimental Model of Lyme Arthritis

**DOI:** 10.3390/diagnostics10100790

**Published:** 2020-10-06

**Authors:** Anne Skovsbo Clausen, Mathilde Ørbæk, Regitze Renee Pedersen, Peter Oestrup Jensen, Anne-Mette Lebech, Andreas Kjaer

**Affiliations:** 1 Department of Clinical Physiology, Nuclear Medicine & PET, Rigshospitalet, DK-2100 Copenhagen, Denmark; anne.clausen@sund.ku.dk; 2 Cluster for Molecular Imaging, Department of Biomedical Sciences, University of Copenhagen, DK-2200 Copenhagen, Denmark; 3 Department of Infectious Diseases, Copenhagen University Hospital, Rigshospitalet, DK-2100 Copenhagen, Denmark; mathilde.daniele.oerbaek.thalund@regionh.dk (M.Ø.); anne-mette.lebech@regionh.dk (A.-M.L.); 4 Costerton Biofilm Center, Department of Immunology and Microbiology, University of Copenhagen, DK-2200 Copenhagen, Denmark; regitze.pedersen@sund.ku.dk (R.R.P.); pojensen@sund.ku.dk (P.O.J.); 5 Department of Clinical Microbiology, Copenhagen University Hospital, Rigshospitalet, DK-2100 Copenhagen, Denmark; 6 Center for Rheumatology and Spine Diseases, Institute for Inflammation Research, Copenhagen University Hospital, Rigshospitalet, DK-2100, Copenhagen, Denmark; 7 Institute of Clinical Medicine, University of Copenhagen, DK-2100 Copenhagen, Denmark

**Keywords:** lyme borreliosis, *borrelia burgdorferi* sensu lato complex, animal model, macrophage, positron emission tomography (PET), ^64^Cu-DOTATATE PET

## Abstract

Macrophages play a key role in the inflammatory response in Lyme arthritis (LA) and could be a target for diagnosing and monitoring active *Borrelia burgdorferi* sensu lato (*Bb*) infection. Therefore, we evaluated the potential of macrophage imaging using ^64^Cu-DOTATATE PET/CT for detection of *Bb* activity in a murine model of LA. LA was established in C3H/HeNRj mice infected with *Bb* B31 strain ML23 pBBE22*luc*. Bioluminescence imaging was performed to detect migration of spirochetes and inflammatory phagocytes to the joints. Three weeks post-infection ^64^Cu-DOTATATE PET/CT imaging was performed at an early (3 h) and late (48 h) time point. Plasma levels of a systemic macrophage marker in plasma CD163 were measured. ^64^Cu-DOTATATE uptake in infected joints was increased at the early (*p* < 0.0001) and late time points (*p* = 0.0005) compared with uptake in non-infected controls. No significant difference in plasma levels of CD163 was measured. ^64^Cu-DOTATATE PET allows for in vivo detection and quantification of LA locally in the joints through non-invasive visualization of macrophages. In contrast, measurement of a systemic macrophage marker in plasma, CD163, did not allow to detect disease. We suggest that ^64^Cu-DOTATATE PET could become a valuable diagnostic tool for in situ detection of *Bb* infection-related inflammation.

## 1. Introduction

Lyme borreliosis (LB) caused by the *Borrelia burgdorferi* sensu lato (*Bb* s.l.) complex is the most prevalent vector-borne infection in Europe and the US with estimates of annual incidence ranging from 10–80 per 100,000 inhabitants. *Bb* s.l. can cause a variety of clinical manifestations including erythema migrans, Lyme neuroborreliosis and Lyme arthritis. The heterogeneity in the clinical expression of human LB is in part explained by different organotropism of the more than 20 species in the *Bb* s.l. complex. *B. burgdorferi* sensu stricto (*Bb* sensu stricto) is especially arthritogenic and causes Lyme arthritis, whereas *B. garinii* and *B. afzelii* cause Lyme neuroborreliosis and dermatoborreliosis, respectively [1,2,3].

A diagnosis of disseminated LB is based on possible exposure to tick bites, compatible clinical symptoms and objective signs in combination with antibody detection [4]. However, seropositivity alone is insufficient to make a diagnosis of active LB, as antibodies may first be detectable after weeks of infection and may persist for years after the infection has been treated. Additionally, direct detection of *Bb* s.l. by culture or molecular methods have a variable and suboptimal sensitivity. Likewise, *Borrelia* DNA can persist for months in the skin or in synovial fluid even after successful treatment, which renders the difficulty for direct detection of *Borrelia* DNA to unequivocally verify an active infection [5,6,7]. Therefore, new methods to monitor disease activity in patients with LB would be of paramount interest to practice precision medicine in these patients and to promote a shift from empirical treatment to personalized medicine with expected improvement in patient outcome.

During *Bb* s.l. infection, the early immune response is characterized by activation of monocytes/macrophages, which is sustained as long as active infection is present [2,8]. Furthermore, increased recruitment and number of macrophages with high phagocytic activity have been found in the joints of *Bb*-infected mice [9] as well as in studies of LB in rhesus macaques [10,11]. Thus, macrophages play a key role in the inflammatory response in Lyme arthritis and could be a potential target for diagnosing, localizing and monitor active inflammation caused by *Bb* infection [12,13]. 

We previously developed the positron emission tomography (PET) tracer ^64^Cu-DOTATATE targeting somatostatin receptor subtype-2 (sstr2), which is known to be overexpressed on activated macrophages both in mice and humans [14,15]. Therefore, ^64^Cu-DOTATATE could potentially serve as non-invasive imaging agent for detection and monitoring of LB. In addition, the long half-life of ^64^Cu of 12.7 h and the high spatial resolution on PET using this isotope make it attractive for clinical routine use. However, macrophages also have significant impact on the inflammatory response in other diseases including rheumatoid arthritis (RA). The collagen-induced arthritis (CIA) mouse model that mimics human autoimmune arthritis is a widely studied animal model of human RA and was in the present study used to confirm tracer uptake in inflamed joints [16]. Moreover, in vivo bioluminescence imaging using lucigenin, which is activated by phagocyte NADPH oxidase in macrophages, was performed [17].

Furthermore, cluster of differentiation (CD) 163, a biomarker of macrophages gene expression, is known to correlate with ^64^Cu-DOTATATE uptake, indicating that ^64^Cu-DOTATATE PET is detecting activated macrophages [18,19]. Moreover, the development of arthritis in CIA mice is dependent on the macrophage biomarker: CD163 [20].

The aim of the present study was to evaluate the potential of macrophage imaging using ^64^Cu-DOTATATE positron emission tomography/computed tomography (PET/CT) for non-invasive in vivo detection and quantification of *Bb* activity in a murine model of Lyme arthritis. In addition, we wanted to test if plasma levels of CD163 could be used to detect *Bb* infection.

## 2. Materials and Methods

### 2.1. Mice

Six-week-old, female C3H/HeNRj and DBA/1 mice were purchased (Janvier, Le Genest-Saint-Isle, France) and housed in groups of 4–8 mice in individually ventilated cages under standardized lighting conditions and fed pathogen-free food and water ad libitum. All animal experiments had been approved by The Danish Working Environment Authority and by The Animal Experiments Inspectorate in Denmark under the license number 2016-15-0201-00920, approved on 5 July 2016.

### 2.2. Induction of Bb Infection

*Bb* B31 strain ML23 was made competent and transformed with the bioluminescent plasmid pBBE22*luc*, and the transformants were selected for resistance to kanamycin. *Bb* B31 strain ML23 pBBE22*luc* was kindly provided by Dr. Jon T. Skare. Cultures were grown in Barbour–Stoenner–Kelly medium (BSK-H) supplemented with 6% rabbit serum (Merck KGaA, Darmstadt, Germany) under conventional microaerobic conditions (1–5% CO_2_, 33 °C) created in Oxoid^TM^ Compact Plastic Pouches (Thermo Fisher Scientific, Waltham, MA, USA) with a OxoidTM Campygen^TM^ Compact sachet (Thermo Fisher Scientific), sealed airtight with a sealing clip [21]. Medium was filter-sterilized with 0.2 μm filter and incubated with kanamycin (300 μg/mL) [22]. After incubation, the cultures were checked with fluorescence microscopy (Axio Imager.Z2, LSM880 CLSM; Zeiss, Jena, Germany) using Syto9 (Molecular Probes, Eugene, OR, USA) emitting a green fluorescent signal to ensure the morphologies of the spirochete before being used for infection. Prior to infection, confocal fluorescence microscopy confirmed the helical shaped morphology of spirochetes. Images were processed using IMARIS 9.2 software (Bitplane, Zurich, Switzerland). Total cell counting of the bacterial culture was performed in duplicate by flow cytometry using a FacsCanto II (BD Biosciences, La Jolla, CA, USA). A volume of 10 μL bacterial solution in 1 mL Dulbecco’s Phosphate Buffered Saline (PBS) (Biowest, Nuaillé, France) was stained with 5 μM Syto9 and added to a TruCount tube (BD Bioscience). Bacteria were discriminated according to their light scatter and Syto9 fluorescence. The counting beads were identified according to their fluorescens at 485-535 nm and above 610 nm. Propidium iodide (Merck KGaA, Darmstadt, Germany) was used for bacterial viability staining as it only enters cells with compromised membranes, binds to DNA and RNA and emits red fluorescence [23]. The bacterial culture was adjusted with PBS to a concentration of 20 × 10^6^ cells/mL. Two setups of C3H/HeNRj mice were anaesthetized (2–3% isoflurane in O_2_) and inoculated intradermally in the lower back with 10^6^ ML23 pBBE22*luc* (50 µL) (*n* = 2 × 16) or with PBS (50 µL) (*n* = 2 × 6). Blood samples (50–100 μL of blood) were collected from *Bb*-infected and PBS control mice by submandibular bleeding using single-use lancet without the use of anesthesia [24]. Blood was centrifuged (1800× *g*, 10 min, 4 °C), and plasma was stored at −80 °C until analysis.

### 2.3. Collagen-Induced Arthritis

DBA/1 mice were immunized with chicken type II collagen (Chondrex, Redmond, WA, USA) emulsified in complete Freund’s adjuvant (Sigma-Aldrich, St. Louis, MO, USA) by subcutaneous injection in the tail (100 μL). After three weeks, mice received a booster immunization with type II collagen emulsified in incomplete Freund’s adjuvant (Sigma-Aldrich) (100 μL) [25]. Mice were anesthetized in 3–4% sevoflurane in 65% N_2_ and 35% O_2_ during both immunizations. Four weeks after initial immunization, signs of arthritis in the paws developed. Each paw was scored three times a week on a scale from 0 to 4. The following criteria were used: 0, normal paw; 1, one toe inflamed and swollen; 2, more than one toe, but not entire paw, inflamed and swollen or mild swelling of entire paw; 3, entire paw inflamed and swollen and 4, very inflamed and swollen paw or ankylosed paw. A mean score of 2.5 for all paws was considered maximum per animal. 

### 2.4. In Vivo Bb Bioluminescence Imaging

Mice were intraperitoneally (ip.) injected with 5 mg D-luciferin (30 mg/mL in PBS) 10 min before bioluminescence imaging using an IVIS Lumina XR imaging system (Caliper Life Sciences, Hopkinton, MA, USA). Luciferase bioluminescence intensities were measured using 120–180 s exposure on the mice in ventral/sternal recumbency followed by 120–180 s exposure in dorsal recumbency (f/stop = 1, emission filter = open and binning = 8). Imaging was performed the 1st day after inoculation and subsequently 1, 2, 3, 4, 5, 6 and 8 weeks after inoculation with *Bb*. Control mice were measured for bioluminescence after injection of D-luciferin 1, 2, 3, 4, 5 and 6 weeks after PBS injection. Additionally, inflammatory bioluminescence activities were detected using lucigenin (bis-N-methylacridinium nitrate, 1.25 mg/mL in isotonic saline solution). Mice were ip. injected with lucigenin (12.5 mg/kg) and imaging was performed 10 min after injection. Lucigenin bioluminescence imaging was performed 3 weeks after inoculation with *Bb*. All images were analyzed using Living Image Software version 4.2 (Caliper Life Sciences, Hopkinton, MA, USA). ROIs were drawn for quantitative measurement of the luminescence (total flux) in photons/second (p/s) using equal ROI size for carpal joints as well as for tarsal joints for all mice, infected and negative controls. The geometrical mean of each carpal or tarsal joint was calculated from both ventral and dorsal ROI quantification. 

### 2.5. Synthesis and Radiolabeling of [^64^Cu]Cu-DOTATATE

The ligand [1,4,7,10-tetraazacyclododecane-*N*,*N*′,*N*″,*N*‴-tetraacetic acid]-*d*-Phe1,Tyr3-octreotate (DOTATATE) was labeled with the long-lived PET isotope ^64^Cu. [^64^Cu]Cu-DOTATATE was produced at DTU Nutech Hevesy Lab, Risør, Denmark, as previously described and approved under good manufacturing practice [26]. 

### 2.6. PET/CT Imaging Acquisition and Analysis 

PET/CT imaging (Inveon, Siemens Medical Systems, Malvern, PA, USA) was performed as a double-bed (two mice at a time), 3 weeks after inoculation with *Bb* (*n* = 16) or PBS for control mice (*n* = 6). Mice were injected intravenously with [^64^Cu]Cu-DOTATATE in a radioactive dose of 10.0 ± 0.2 MBq (mean ± SD). After injection, mice were returned to cages to rest before PET/CT scan. Animals were anesthetized (3–5% sevoflurane in 35% O_2_/65% N_2_) and PET imaging was performed 3 h (termed early scan, 300 s PET acquisition) and 48 h (termed late scan, 900 s PET acquisition) after [^64^Cu]Cu-DOTATATE injection. CT was performed for anatomical reference and for attenuation correction. Emission data were corrected for dead time and decay. PET scans were reconstructed with the maximum a posterior reconstruction algorithm. Inveon Research Workstation software (Siemens Medical Systems, PA, USA) was used for quantitative analysis. ROIs were manually drawn on the carpal and tarsal joints of each mouse, using CT as an anatomical guide. The uptake of [^64^Cu]Cu-DOTATATE was quantified as maximal percentage of injected dose per gram tissue (%Max ID/g)

### 2.7. CD163 Enzyme-Linked Immunosorbent Assay

Murine CD163 concentrations were measured in 200-fold diluted plasma by CD163 ELISA Kit (antibodies-online GmbH, Aachen, Germany) according to the manufacturer’s instructions. The absorbance was measured at 450 nm with a FLUOstar Omega plate reader (BMG Labtech, Ortenberg, Germany) and subtracted with a reference value measured at 650 nm. The concentration of CD163 was calculated using a standard curve and the measured absorbance. Values above the standard curve was reported as 100 ng/mL.

### 2.8. Statistical Analysis

Unpaired t-test was used for comparisons of lucigenin total flux between *Bb*-infected and control mice, ^64^Cu-DOTATATE uptake between early (3 h) and late scan (48 h) and CD163 plasma levels between *Bb*-infected and control mice at weeks 2, 3, 4 and 5 post-infection. A *p* value of less than 0.05 was considered statistically significant. Values are reported as mean ± SEM unless otherwise stated. Prism version 7.00 (GraphPad, San Diego, CA, USA) was used for all statistical analyses. 

## 3. Results

### 3.1. Bb Infection and Migration of Spirochetes to the Joints

**Figure 1:**
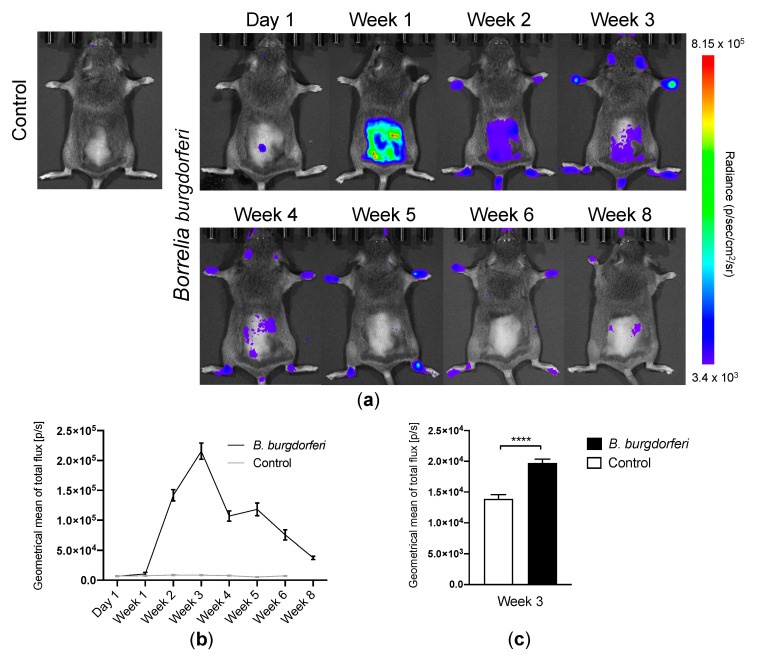
In vivo bioluminescence imaging of *B. burgdorferi* infection and inflammatory phagocytes: (**a**) Representative images of a control mouse and *Bb*-infected mice at day 1 followed by 1, 2, 3, 4, 5, 6 and 8 weeks after intradermal inoculation with *B. burgdorferi* strain ML23 pBBE22*luc*. Images were acquired following ip. injection of D-luciferin. (**b**) Geometrical mean of total flux (p/s) of mice after ip. injection of D-luciferin. In vivo imaging was performed on *Bb*-infected (*n* = 6–26/group) and control (*n* = 4–10/group) mice at day 1 followed by week 1, 2, 3, 4, 5, 6 and 8 after intradermal inoculation. All values are derived from ROI analysis of carpal and tarsal joints. Data are presented as mean ± SEM. (**c**) Quantitative representation of lucigenin bioluminescence from the ROI analysis of the carpal and tarsal joints after ip. injection of lucigenin (12.5 mg/kg) in *Bb*-infected (*n* = 18) and control (*n* = 6) mice on week 3 after inoculation. Data are presented as mean ± SEM. All images were acquired with a delay of 10 min post-injection (p.i.) of substrate before imaging was completed. The significance level is indicated by asterisks (*). *p* < 0.0001 (****).

All *Bb* injected mice became successfully infected. Bioluminescence imaging detected *Bb*-infected mice, which showed focal bioluminescent signal centrally on the lower back at the initial imaging time point (day 1) post-infection at the site of injection. This local infection induced by injection of ML23 pBBE22*luc* strain of *Bb* B31 expanded in the mouse presumably due to dissemination throughout the skin, which was observed one week post-infection as an intensive signal from the dorsal surface of the mice. Two weeks post-infection, the emitted signal spread in the mice, and the bioluminescent signal appeared at the extremities (Figure 1a). All mice were imaged in ventral and dorsal recumbency at each time point. Based on these values, the geometrical mean of the total flux in each joint was calculated as a measure for total amount of bacteria per joint. A peak in the bioluminescence emission from each carpal and tarsal joint was observed 3 weeks post-infection compared with the control mice. Starting three weeks after inoculation, the signal started to diminish but remained visible until the last day of imaging (Figure 1b). Eight weeks after infection, the bioluminescent signal from the joints reached low geometrical means representing disappearance of *Bb.* Bioluminescence signals from the joints were 1.1 × 10^4^ ± 2.5 × 10^3^, 1.4 × 10^5^ ± 9.5 × 10^3^, 2.2 × 10^5^ ± 1.4 × 10^4^, 1.1 × 10^5^ ± 8.7 × 10^3^, 1.2 × 10^5^ ± 1.1 × 10^4^, 7.5 × 10^4^ ± 8.6 × 10^3^, 3.7 × 10^4^ ± 2.8 × 10^3^ p/s for the 1, 2, 3, 4, 5, 6 and 8 week time points, respectively. Quantitative data on lucigenin bioluminescence imaging 3 weeks post-infection are depicted in Figure 1c, and the average geometrical mean of the total flux was 2.0 × 10^4^ ± 6.3 × 10^2^ and 1.4 × 10^4^ ± 7.0 × 10^2^ for *Bb*-infected and control mice, respectively. Total flux (p/s) was significantly increased in *Bb*-infected mice 3 weeks post-infection compared with control mice (*p* < 0.0001).

### 3.2. [^64^Cu]Cu-DOTATATE PET/CT Imaging of Macrophages in CIA and Lyme Arthritis

**Figure 2:**
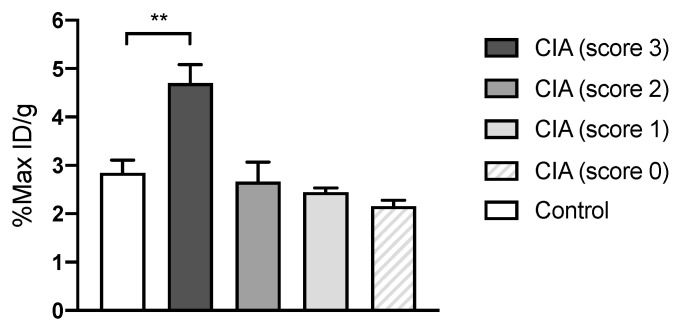
In vivo quantification of ^64^Cu-DOTATATE uptake in collagen-induced arthritis (CIA) mice (*n* = 7) and controls (*n* = 4). Data are presented as maximal percentage of injected dose per gram tissue (%Max ID/g) in each joint acquired 3 h p.i. Data are presented as mean ± SEM. The significance level is indicated by asterisks (*). *p* = 0.0032 (**).

In addition to PET imaging of Lyme arthritis in *Bb* mice, ^64^Cu-DOTATATE was tested in the inflammatory CIA mouse model. Quantitative data on the uptake in joints are depicted in Figure 2. The maximum ^64^Cu-DOTATATE uptake in joints with high CIA arthritis score (score 3) was 4.70% ± 0.38% 3 h p.i. Maximum tracer uptake was significantly increased in these joints compared with control mice (*p* = 0.0032). Maximum uptake in control mice was 2.85% ± 0.26% 3 h p.i.

**Figure 3:**
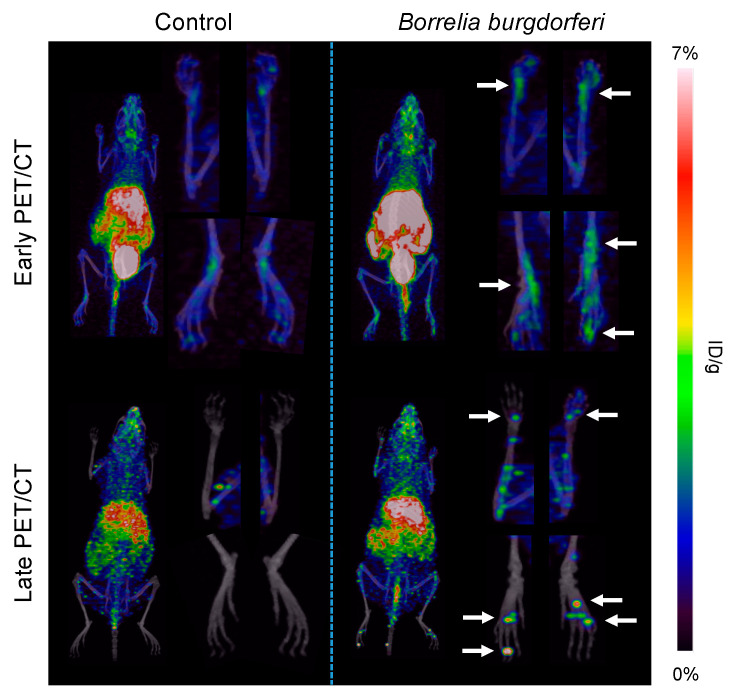
^64^Cu-DOTATATE coronal PET/CT imaging of *Bb*-infected and control mice. Representative images of mice inoculated intradermally with *B. burgdorferi* (*n* = 18) or PBS (*n* = 6). Images were acquired 3 weeks after inoculation at 3 h (early PET/CT) and 48 h (late PET/CT) p.i. Images of the ROIs drawn around the carpal and tarsal joints are shown next to the whole-body images. Arrows designate high tracer uptake in the carpal and tarsal joints.

**Figure 4:**
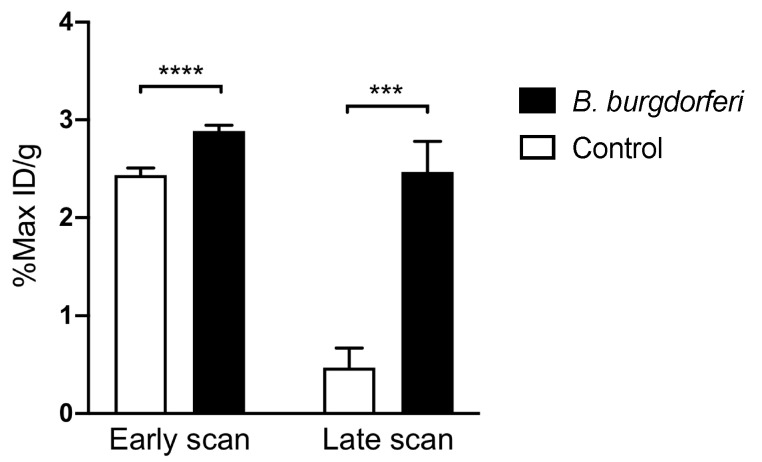
In vivo quantification of accumulated ^64^Cu-DOTATATE in mice inoculated with *B. burgdorferi* (*n* = 18) or PBS (*n* = 6). Data are presented as maximal percentage of injected dose per gram tissue (%Max ID/g) in each joint and acquired 3 h (early scan) and 48 h (late scan) p.i. 3 weeks post-infection. Data are presented as mean ± SEM. The significance level is indicated by asterisks (*). *p* = 0.0005 (***) and *p* < 0.0001 (****).

In vivo PET/CT imaging of *Bb*-induced Lyme arthritis 3 weeks post-infection in mice showed an increased, intense focal accumulation of ^64^Cu-DOTATATE in the joints at the late scan (48 h p.i. of PET tracer) compared with uninfected control mice. Images acquired 3 h p.i. of tracer displayed a more diffuse uptake within the carpal and tarsal joints of the infected mice. Control mice showed little to no uptake in the joints (Figure 3). Quantitative data on uptake in *Bb*-infected joints are illustrated in Figure 4, and the maximum ^64^Cu-DOTATATE uptake in infected joints (%Max ID/g) was 2.89% ± 0.06% 3 h p.i. (early scan) and 2.47% ± 0.31% 48 h p.i. (late scan). Maximum tracer uptake was significantly increased in the infected joints at the early (*p* < 0.0001) and late scan time point (*p* = 0.0005) compared with the uptake in control mice. Maximum uptake in control mice was 2.44% ± 0.07% 3 h p.i. (early scan) and 0.47% ± 0.20% 48 h p.i. (late scan) (Figure 4). 

### 3.3. CD163 as a Circulating Marker for Macrophages

**Figure 5:**
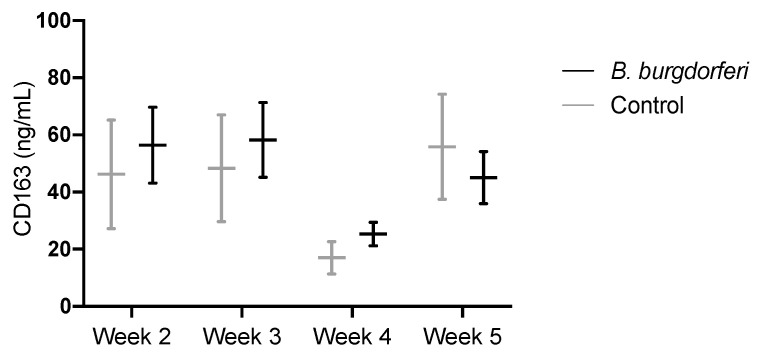
CD163 plasma levels (ng/mL) in a murine model of Lyme arthritis. Mice were infected with 10^6^ *B. burgdorferi* intradermally in the lower back, and cheek blood was collected at week 2, 3, 4 (*n* = 8) and 5 (*n* = 15) post-infection. Data are presented as mean ± SEM.

Plasma levels of CD163 in infected mice were 56.4 ± 13.3, 58.3 ± 13.1, 25.3 ± 4.1 and 45.0 ± 9.1 ng/mL for the 2-, 3-, 4- and 5-week time points, respectively. No significant differences between *Bb*-infected and control mice were observed at any of the time points (Figure 5). In addition, samples from individual mice showed heterogeneous levels of CD163. Variations at each time point for both *Bb*-infected and control mice appeared, and 23.6% of all samples were higher than 100 ng/mL.

## 4. Discussion

The major finding in the present study was that PET/CT imaging using ^64^Cu-DOTATATE, a PET tracer demonstrating macrophages, could detect and monitor Lyme arthritis activity non-invasively, whereas levels of CD163, a marker of macrophages in the plasma during bacterial infection, could not be used for this purpose. 

Using bioluminescence, we showed that ML23 pBBE22*luc* strain of *Bb* B31 administered intradermally migrated to the joints in the mice, and a peak of bacterial burden in the joints was observed 3 weeks after inoculation of the spirochetes representing direct evidence of pathogens within the joints. The stage of the joint invasion by *Bb* in this model represents the early phase of Lyme arthritis. Bioluminescence imaging also confirmed the presence of macrophages in Lyme arthritis lesions in the infected mice. 

This is the first study to present data on somatostatin receptor imaging of infectious lesions in LB. ^64^Cu-DOTATATE is mainly being used in the clinical setting for neuroendocrine tumors (NETs) [18,26,27] but has also been tested for detection of atherosclerotic inflammation [19]. In NETs, the basis for imaging is overexpression of somatostatin receptors on the tumors cells [28] whereas the basis for use in atherosclerosis is the macrophages in the atherosclerotic lesions [19,29]. Since *Bb* infection in the joints is likely to be characterized by macrophage invasion [12], we hypothesized that ^64^Cu-DOTATATE could be a useful tool for diagnosis and monitoring of Lyme arthritis by imaging of macrophages. 

Before testing our hypothesis, we showed that ^64^Cu-DOTATATE can be used in another inflammatory model as the tracer significantly accumulates in the joints of CIA mice with high clinical score at an early time point. Data from this study confirmed that the tracer can be used to target a local inflammatory response and allowed us to continue the work with ^64^Cu-DOTATATE on our *Bb*-infection model.

^64^Cu has a half-life of 12.7 h giving the possibility for delayed imaging, and therefore, we tested two different time points for PET scanning, an early (3 h) and a late (48 h) after tracer injection. Our results indicate that PET imaging using ^64^Cu-DOTATATE of Lyme arthritis in mice should be performed after 48 h to enable clearance of background activity. We showed a significantly increased uptake of tracer (%Max ID/g) in infected mice imaged at the late time point (48 h). 

Currently, the most commonly used tracer in PET imaging for infection and inflammation is fluorine-18-fluorodeoxyglucose (^18^F-FDG), a radiolabeled glucose analogue [30,31,32]. However, ^18^F-FDG has major limitations including being non-specific for infection. FDG is taken up by cells via glucose transporters in the membrane, and many different cells are involved including leukocytes, macrophages, monocytes, lymphocytes and giant cells [33]. Due to these limitations, there is an unmet need for other, more specific, targeted radiotracers to improve imaging of infectious diseases and the adjacent inflammatory response. Only few other PET tracers have previously been tested to visualize the inflammatory response caused by *Bb.* Pietikäinen et al. tested ^18^F-FDG [31] and showed increased uptake in affected joints. However, ^18^F-FDG has limitations as previously described. Siitonen et al. tested ^68^Ga-DOTA-Siglec-9 a PET tracer targeting vascular adhesion protein-1 (VAP-1) and showed uptake in infected joints. Both tracers where only tested in tibiotarsal joints in a Lyme arthritis murine model.

In our model, we utilized direct intradermal inoculation of bacteria that allowed bacteria to enter the bloodstream mimicking the pathogenesis in human Lyme arthritis although not transmitted by a vector. The C3H/HeN mouse is the most frequently used animal strain to investigate the immune mechanisms of Lyme arthritis. The model has the advantages of mimicking the early innate immune response that occurs when the bacteria enter the host when transmitted by a vector [34], and using C3H/HeN mice is a promising model for studying the inflammation of subacute Lyme arthritis as a response to spirochetes localized in the joint tissue [35]. This allow us to test our ^64^Cu-DOTATATE PET tracer as an early marker for the bacterial infection. However, using bioluminescence, we showed that C3H/HeN mice cleared the *Bb* infection in the joints 3 to 6 weeks after inoculation, which is not consistent with human Lyme arthritis, and the model does not mimic the immunological events that result in severe arthritis. We know that our model displays mild and not sustained symptoms of arthritis indicating that the inflammatory response is mild, which makes it more difficult to show increased accumulation of ^64^Cu-DOTATATE. Having this in mind, our findings on increased accumulation of the tracer within the joints of *Bb-*infected mice seem promising as an early marker for Lyme arthritis in the patients and have great potential in the clinic. In addition, previous studies have described that macrophages play a direct effector role in the induction of severe destructive Lyme arthritis [36], substantiating the clinical use of ^64^Cu-DOTATATE as a biomarker for macrophages in the joints. Potentially, the severity of Lyme arthritis in regard to immune infiltration could be used in the clinic for initial treatment regimens. However, when using the tracer, it should be kept in mind that it is not specific for Lyme arthritis, and other inflammatory joint conditions may appear similar.

We previously showed that ^64^Cu-DOTATATE is significantly associated with biomarkers of CD163 positive macrophage activation when studied in atherosclerotic patients [19]. This clearly demonstrates the systemic nature of atherosclerosis with inflammation in the vasculature. In contrast, the present study was not able to show any increased level of CD163 in the plasma during infection with *Bb* in mice. This might not be surprising as Lyme arthritis has a localized nature. Therefore, it seems CD163 cannot be used as a circulatory biomarker for Lyme arthritis as these results show no systemic upregulation of CD163^+^ macrophages. Based on these results, we must assume that Lyme arthritis results in a localized macrophage response within the joint and not a systemically induced response in the blood of C3H/HeN mice. These results highlight the necessity and benefit of non-invasive in vivo imaging; local detection of the inflammatory response as seen in Lyme arthritis may be demonstrated. The reason for testing circulating CD163 was that this would constitute an easy method in the clinics. However, it did not turn out to be of value in our study.

In conclusion, ^64^Cu-DOTATATE PET allows for detection of LB infection locally in the joints through non-invasive visualization of macrophages. In contrast, measurement of a systemic macrophage marker in plasma, CD163, did not allow to detect disease. We suggest, that ^64^Cu-DOTATATE PET could become a valuable diagnostic tool for in situ detection of *Bb* infection-related inflammation.

## References

[1] Stanek G., Strle F. (2018). Lyme borreliosis-from tick bite to diagnosis and treatment. FEMS Microbiol. Rev..

[2] Steere A.C., Strle F., Wormser G.P., Hu L.T., Branda J.A., Hovius J.W., Li X., Mead P.S. (2016). Lyme borreliosis. Nat. Rev. Dis. Primers.

[3] Steere A.C., Glickstein L. (2004). Elucidation of Lyme arthritis. Nat. Rev. Immunol..

[4] Dessau R.B., van Dam A.P., Fingerle V., Gray J., Hovius J.W., Hunfeld K.P., Jaulhac B., Kahl O., Kristoferitsch W., Lindgren P.E. (2018). To test or not to test? Laboratory support for the diagnosis of Lyme borreliosis: A position paper of ESGBOR, the ESCMID study group for Lyme borreliosis. Clin. Microbiol. Infect..

[5] Obel N., Dessau R.B., Krogfelt K.A., Bodilsen J., Andersen N.S., Moller J.K., Roed C., Omland L.H., Christiansen C.B., Ellermann-Eriksen S. (2018). Long term survival, health, social functioning, and education in patients with European Lyme neuroborreliosis: Nationwide population based cohort study. BMJ.

[6] Raffetin A., Saunier A., Bouiller K., Caraux-Paz P., Eldin C., Gallien S., Jouenne R., Belkacem A., Salomon J., Patey O. (2020). Unconventional diagnostic tests for Lyme borreliosis: A systematic review. Clin. Microbiol. Infect..

[7] Shapiro E.D., Wormser G.P. (2018). Lyme Disease in 2018: What Is New (and What Is Not). JAMA.

[8] Strle K., Drouin E.E., Shen S., El Khoury J., McHugh G., Ruzic-Sabljic E., Strle F., Steere A.C. (2009). Borrelia burgdorferi stimulates macrophages to secrete higher levels of cytokines and chemokines than Borrelia afzelii or Borrelia garinii. J. Infect. Dis..

[9] Lasky C.E., Olson R.M., Brown C.R. (2015). Macrophage Polarization during Murine Lyme Borreliosis. Infect Immun..

[10] Ramesh G., Didier P.J., England J.D., Santana-Gould L., Doyle-Meyers L.A., Martin D.S., Jacobs M.B., Philipp M.T. (2015). Inflammation in the pathogenesis of lyme neuroborreliosis. Am. J. Pathol..

[11] Eiffert H., Karsten A., Schlott T., Ohlenbusch A., Laskawi R., Hoppert M., Christen H.J. (2004). Acute peripheral facial palsy in Lyme disease—A distal neuritis at the infection site. Neuropediatrics.

[12] Wooten R.M., Weis J.J. (2001). Host-pathogen interactions promoting inflammatory Lyme arthritis: Use of mouse models for dissection of disease processes. Curr. Opin. Microbiol..

[13] Xu Q., Seemanapalli S.V., Reif K.E., Brown C.R., Liang F.T. (2007). Increasing the recruitment of neutrophils to the site of infection dramatically attenuates Borrelia burgdorferi infectivity. J. Immunol..

[14] Dalm V.A., van Hagen P.M., van Koetsveld P.M., Achilefu S., Houtsmuller A.B., Pols D.H., van der Lely A.J., Lamberts S.W., Hofland L.J. (2003). Expression of somatostatin, cortistatin, and somatostatin receptors in human monocytes, macrophages, and dendritic cells. Am. J. Physiol. Endocrinol. Metab..

[15] Perez J., Viollet C., Doublier S., Videau C., Epelbaum J., Baud L. (2003). Somatostatin binds to murine macrophages through two distinct subsets of receptors. J. Neuroimmunol..

[16] Courtenay J.S., Dallman M.J., Dayan A.D., Martin A., Mosedale B. (1980). Immunisation against heterologous type II collagen induces arthritis in mice. Nature.

[17] Tseng J.C., Kung A.L. (2013). In vivo imaging method to distinguish acute and chronic inflammation. J. Vis. Exp..

[18] Johnbeck C.B., Knigge U., Loft A., Berthelsen A.K., Mortensen J., Oturai P., Langer S.W., Elema D.R., Kjaer A. (2017). Head-to-Head Comparison of (64)Cu-DOTATATE and (68)Ga-DOTATOC PET/CT: A Prospective Study of 59 Patients with Neuroendocrine Tumors. J. Nucl. Med..

[19] Pedersen S.F., Sandholt B.V., Keller S.H., Hansen A.E., Clemmensen A.E., Sillesen H., Hojgaard L., Ripa R.S., Kjaer A. (2015). 64Cu-DOTATATE PET/MRI for Detection of Activated Macrophages in Carotid Atherosclerotic Plaques: Studies in Patients Undergoing Endarterectomy. Arter. Thromb. Vasc. Boil..

[20] Svendsen P., Etzerodt A., Deleuran B.W., Moestrup S.K. (2020). Mouse CD163 deficiency strongly enhances experimental collagen-induced arthritis. Sci. Rep..

[21] Hubalek Z., Halouzka J., Heroldova M. (1998). Growth temperature ranges of Borrelia burgdorferi sensu lato strains. J. Med. Microbiol..

[22] Hyde J.A., Weening E.H., Chang M., Trzeciakowski J.P., Hook M., Cirillo J.D., Skare J.T. (2011). Bioluminescent imaging of Borrelia burgdorferi in vivo demonstrates that the fibronectin-binding protein BBK32 is required for optimal infectivity. Mol. Microbiol..

[23] Rosenberg M., Azevedo N.F., Ivask A. (2019). Propidium iodide staining underestimates viability of adherent bacterial cells. Sci. Rep..

[24] Golde W.T., Gollobin P., Rodriguez L.L. (2005). A rapid, simple, and humane method for submandibular bleeding of mice using a lancet. Lab. Anim. (NY).

[25] Pietrosimone K.M., Jin M., Poston B., Liu P. (2015). Collagen-Induced Arthritis: A model for Murine Autoimmune Arthritis. Bio-Protocol.

[26] Pfeifer A., Knigge U., Mortensen J., Oturai P., Berthelsen A.K., Loft A., Binderup T., Rasmussen P., Elema D., Klausen T.L. (2012). Clinical PET of neuroendocrine tumors using 64Cu-DOTATATE: First-in-humans study. J. Nucl. Med..

[27] Kjaer A., Binderup T., Johnbeck C., Carlsen E., Langer S., Federspiel B., Knigge U. (2019). Cu-64-DOTATATE somatostatin receptor Imaging in neuroendocrine tumors: Experience from 500 patients at Copenhagen ENETS Center of Excellence. J. Nucl. Med..

[28] Binderup T., Knigge U., Loft A., Mortensen J., Pfeifer A., Federspiel B., Hansen C.P., Hojgaard L., Kjaer A. (2010). Functional imaging of neuroendocrine tumors: A head-to-head comparison of somatostatin receptor scintigraphy, 123I-MIBG scintigraphy, and 18F-FDG PET. J. Nucl. Med..

[29] Pedersen S.F., Graebe M., Hag A.M., Hojgaard L., Sillesen H., Kjaer A. (2013). (18)F-FDG imaging of human atherosclerotic carotid plaques reflects gene expression of the key hypoxia marker HIF-1alpha. Am. J. Nucl. Med. Mol. Imaging.

[30] Jamar F., Buscombe J., Chiti A., Christian P.E., Delbeke D., Donohoe K.J., Israel O., Martin-Comin J., Signore A. (2013). EANM/SNMMI guideline for 18F-FDG use in inflammation and infection. J. Nucl. Med..

[31] Pietikainen A., Siitonen R., Liljenback H., Eskola O., Soderstrom M., Roivainen A., Hytonen J. (2018). In vivo imaging of Lyme arthritis in mice by [(18)F]fluorodeoxyglucose positron emission tomography/computed tomography. Scand. J. Rheumatol..

[32] Vaidyanathan S., Patel C.N., Scarsbrook A.F., Chowdhury F.U. (2015). FDG PET/CT in infection and inflammation--current and emerging clinical applications. Clin. Radiol..

[33] Glaudemans A.W., Slart R.H., van Dijl J.M., van Oosten M., van Dam G.M. (2015). Molecular imaging of infectious and inflammatory diseases: A terra incognita. J. Nucl. Med..

[34] Munson E., Nardelli D.T., Du Chateau B.K., Callister S.M., Schell R.F. (2012). Hamster and murine models of severe destructive Lyme arthritis. Clin. Dev. Immunol..

[35] Barthold S.W., Beck D.S., Hansen G.M., Terwilliger G.A., Moody K.D. (1990). Lyme borreliosis in selected strains and ages of laboratory mice. J. Infect. Dis..

[36] Du Chateau B.K., England D.M., Callister S.M., Lim L.C., Lovrich S.D., Schell R.F. (1996). Macrophages exposed to Borrelia burgdorferi induce Lyme arthritis in hamsters. Infect. Immun..

